# Clinical applications of STING agonists in cancer immunotherapy: current progress and future prospects

**DOI:** 10.3389/fimmu.2024.1485546

**Published:** 2024-10-02

**Authors:** Bin Wang, Wanpeng Yu, Hongfei Jiang, Xiangwei Meng, Dongmei Tang, Dan Liu

**Affiliations:** ^1^ The Afffliated Hospital of Qingdao University, Qingdao University, Qingdao, China; ^2^ Medical Education Department, Guangdong Provincial People’s Hospital, Zhuhai Hospital (Jinwan Central Hospital of Zhuhai), Zhuhai, China; ^3^ Department of Drug Clinical Trials, Zibo Central Hospital, Zibo, China; ^4^ Department of Anesthesia, Affiliated Hospital of Qingdao University, Qingdao, China

**Keywords:** STING agonists, cancer immunotherapy, innate immunity, cGAS-STING pathway, tumor microenvironment

## Abstract

The STING (Stimulator of Interferon Genes) pathway is pivotal in activating innate immunity, making it a promising target for cancer immunotherapy. STING agonists have shown potential in enhancing immune responses, particularly in tumors resistant to traditional therapies. This scholarly review examines the diverse categories of STING agonists, encompassing CDN analogues, non-CDN chemotypes, CDN-infused exosomes, engineered bacterial vectors, and hybrid structures of small molecules-nucleic acids. We highlight their mechanisms, clinical trial progress, and therapeutic outcomes. While these agents offer significant promise, challenges such as toxicity, tumor heterogeneity, and delivery methods remain obstacles to their broader clinical use. Ongoing research and innovation are essential to overcoming these hurdles. STING agonists could play a transformative role in cancer treatment, particularly for patients with hard-to-treat malignancies, by harnessing the body’s immune system to target and eliminate cancer cells.

## 1 Introduction

The cyclic GMP-AMP synthase-stimulator of interferon genes (cGAS-STING) pathway is a critical component of the innate immune system, playing a pivotal role in the detection of cytosolic DNA and the subsequent activation of an immune response (1–5). This pathway is evolutionarily conserved and acts as a crucial defense mechanism against pathogens, as well as a mediator of autoimmune responses (6–8). Upon detection of cytosolic DNA, typically from viral or bacterial pathogens, the cGAS enzyme synthesizes cyclic GMP-AMP (cGAMP), a second messenger that directly activates the STING receptor located on the endoplasmic reticulum membrane (9–11). Once activated, STING initiates a cascade of signaling events leading to the production of type I interferons (IFNs) and NF-kB driven pro-inflammatory cytokines, such as TNF-α and IL-6, which are essential for mounting an effective immune response (12).

In the context of cancer, the STING pathway has garnered significant attention due to its ability to induce a potent anti-tumor immune response (13). Tumor cells often harbor aberrant DNA that can activate the cGAS-STING pathway, thereby promoting the production of cytokines and chemokines that recruit and activate immune cells within the tumor microenvironment (14–16). This immunostimulatory effect of STING has positioned it as a promising target for cancer immunotherapy. STING agonists, which are molecules designed to activate the STING pathway, have shown potential in enhancing the immune system’s ability to recognize and destroy tumor cells (17–20). These agonists work by mimicking the natural ligands of STING, thereby amplifying the immune response against tumors.

Given the critical role of STING in immune surveillance and its emerging relevance in cancer therapy, STING agonists have been the subject of intense research (21). Several STING agonists have entered clinical trials, with many showing promising results in enhancing the efficacy of existing immunotherapies, such as immune checkpoint inhibitors (22). These developments highlight the potential of STING agonists to overcome resistance to conventional therapies and to improve outcomes in patients with various types of cancer.

The objective of this review is to provide a comprehensive overview of the current progress in the clinical application of STING agonists in cancer immunotherapy. This review will explore the mechanisms by which STING agonists enhance anti-tumor immunity, summarize the various types of STING agonists currently in clinical development, and discuss the clinical outcomes observed in trials to date. Through this review, we aim to highlight the therapeutic potential of STING agonists in cancer immunotherapy and to discuss their future prospects as a cornerstone of cancer treatment. As research continues to evolve, it is anticipated that STING agonists will play an increasingly important role in the development of novel cancer therapies, offering new hope for patients with difficult-to-treat malignancies.

## 2 Mechanism of STING activation

The STING (Stimulator of Interferon Genes) pathway is a central player in the innate immune system, responsible for detecting cytosolic DNA from various sources, such as viruses, bacteria, and even damaged host cells (23). This pathway initiates a potent immune response that is critical for antiviral defense and has emerging importance in cancer therapy.

### 2.1 Detection and activation

**Figure 1:**
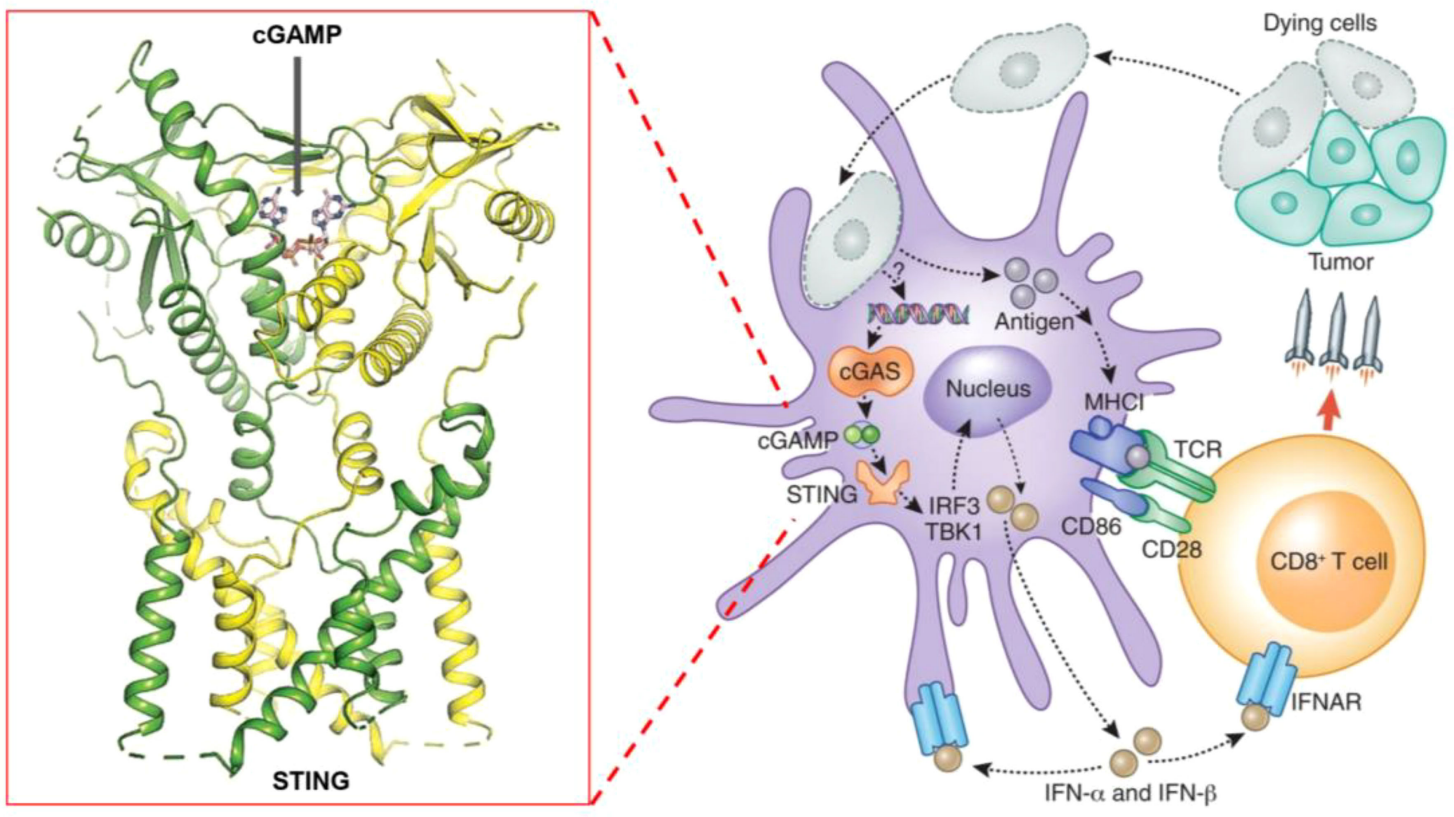
Schematic representation of the STING pathway and its role in cancer immunotherapy, highlighting key steps of activation, downstream signaling, and therapeutic targets.

The STING pathway begins with the recognition of cytosolic DNA by the cGAS (cyclic GMP-AMP synthase) enzyme. Upon detecting double-stranded DNA in the cytosol, cGAS catalyzes the synthesis of cyclic GMP-AMP (cGAMP), a cyclic dinucleotide that serves as a secondary messenger (**Figure 1**). cGAMP then binds directly to the STING protein located on the membrane of the endoplasmic reticulum (ER). This binding triggers a conformational change in STING, leading to its activation (24, 25).

### 2.2 Downstream signaling

Activated STING translocates from the ER to the Golgi apparatus, where it recruits and activates TBK1 (TANK-binding kinase 1) (26). TBK1, in turn, phosphorylates the transcription factor IRF3 (Interferon Regulatory Factor 3) (27). Phosphorylated IRF3 dimerizes and translocates to the nucleus, where it induces the expression of type I interferons and other pro-inflammatory cytokines. These cytokines play a crucial role in recruiting and activating various immune cells, such as dendritic cells, natural killer cells, and T cells, within the tumor microenvironment.

### 2.3 Immune response and tumor suppression

The production of type I interferons and cytokines triggers a robust immune response, enhancing the body’s ability to recognize and eliminate tumor cells (28). Additionally, the activation of STING can lead to the upregulation of immune checkpoint molecules, making tumors more susceptible to immunotherapies like checkpoint inhibitors.

**Figure 1** schematically represents these key steps in the STING pathway, highlighting the crucial role of cGAMP in STING activation, the subsequent signaling cascade, and the production of immune mediators. By understanding these mechanisms, researchers have developed STING agonists that mimic natural ligands like cGAMP, aiming to harness this pathway for cancer immunotherapy.

## 3 Categories of STING agonists

In this section, we explore the diverse categories of STING agonists currently in development for cancer immunotherapy. These agonists can be broadly classified into several types based on their molecular structure and mechanism of action: cyclic dinucleotide (CDN) analogs, which mimic natural cyclic dinucleotides to directly activate STING; non-CDN STING agonists, which trigger STING activation through alternative mechanisms; CDN-loaded exosomes, which use vesicles to deliver STING agonists more efficiently; engineered bacteria vectors that produce STING-activating molecules in situ; small molecule–nucleic acid hybrids that combine stability with specificity; and some undisclosed types whose structures or mechanisms are not fully revealed. Each category represents a unique approach to harnessing the STING pathway, offering different advantages and challenges in clinical application.

### 3.1 CDN analogs

CDN analogs are synthetic molecules that mimic the natural cyclic dinucleotides (such as cGAMP) involved in activating the STING pathway (29, 30). These analogs directly bind to the STING receptor, initiating a cascade of immune responses crucial for anti-tumor activity (31). CDN analogs have shown significant promise in early clinical trials, particularly in combination with immune checkpoint inhibitors.

#### 3.1.1 MK-1454

MK-1454 is a CDN analog developed by Merck, currently in Phase I/II clinical trials (ClinicalTrials.gov-NCT03010176). It is being evaluated for its efficacy in treating solid tumors and lymphomas. In early studies, MK-1454 has been administered intratumorally, and when combined with the anti-PD-1 antibody pembrolizumab, it has demonstrated a favorable safety profile and potential efficacy. Preliminary results indicate that MK-1454 can induce strong immune responses, enhancing the effectiveness of pembrolizumab in shrinking tumors. This drug specifically targets the STING pathway by binding to the STING receptor, leading to the activation of type I interferons and other pro-inflammatory cytokines, which play a crucial role in anti-tumor immunity. Moreover, the localized administration helps to minimize systemic side effects while maximizing immune activation within the tumor microenvironment. The clinical outcomes from these trials suggest that MK-1454 may overcome resistance to PD-1 blockade in some patients (32, 33).

#### 3.1.2 ADU-S100 (MIW815)

ADU-S100, also known as MIW815, is another CDN analog that has been the focus of several clinical studies (ClinicalTrials.gov-NCT02675439, NCT03172936, NCT03937141). Developed by Aduro Biotech and Novartis, ADU-S100 has been evaluated in combination with spartalizumab, an anti-PD-1 therapy. In Phase I/II trials, ADU-S100 is administered intratumorally in patients with solid tumors and lymphomas. The results have shown that ADU-S100, when used in conjunction with spartalizumab, can significantly enhance immune responses, leading to tumor regression in some cases. The combination therapy has been particularly effective in generating a localized immune response, which may help control tumor growth and spread (34, 35).

#### 3.1.3 BMS-986301

BMS-986301 is a CDN analog developed by Bristol-Myers Squibb, currently in Phase I clinical trials for advanced cancers (ClinicalTrials.gov-NCT03956680, NCT03843359). Although detailed clinical data is still emerging, early findings suggest that BMS-986301 has the potential to activate the STING pathway effectively, leading to enhanced anti-tumor immunity. The ongoing trials aim to determine the safety and optimal dosing of BMS-986301, as well as its efficacy in combination with other immunotherapies, such as nivolumab (anti-PD-1) and ipilimumab (anti-CTLA-4) (36).

#### 3.1.4 BI-1387446

BI-1387446 is a CDN analog in development by Boehringer Ingelheim, also in Phase I trials (ClinicalTrials.gov-NCT04147234). This STING agonist is being studied for its use in advanced cancers, and early data indicates that it might be administered in combination with Ezabenlimab, an anti-PD-L1 therapy. While specific clinical outcomes have not yet been fully disclosed, the trials focus on evaluating the safety, tolerability, and preliminary efficacy of BI-1387446 in inducing an immune response against tumors (37).

#### 3.1.5 TAK-676

TAK-676 is a CDN analog being developed by Takeda Oncology, currently in Phase I/II clinical trials (ClinicalTrials.gov-NCT04420884, NCT04879849). This STING agonist is administered intravenously, either alone or in combination with pembrolizumab, in patients with advanced solid tumors and lymphomas. Preliminary results from these trials indicate that TAK-676 is well-tolerated and may potentiate the effects of pembrolizumab, particularly in tumors that are otherwise resistant to immune checkpoint blockade. Ongoing studies are focused on determining the optimal dosing and combination strategies to maximize the therapeutic benefits of TAK-676 (38–40).

### 3.2 Non-CDN chemotypes

Non-CDN STING agonists represent a diverse group of molecules that activate the STING pathway through mechanisms distinct from those of cyclic dinucleotides (CDNs). These agonists often have unique chemical structures that allow them to bind and activate STING in different ways, offering alternative therapeutic strategies for cancer treatment. Below are some of the key non-CDN STING agonists currently in clinical development.

#### 3.2.1 SNX281

SNX281 is a non-CDN STING agonist developed by Silicon Therapeutics, now part of Roivant Sciences. It is currently in Phase I clinical trials for advanced solid tumors (ClinicalTrials.gov-NCT04609579). Unlike CDNs, SNX281 is a small molecule that activates STING without mimicking the natural dinucleotides. The clinical trials are evaluating the safety, tolerability, and preliminary efficacy of SNX281 both as a monotherapy and in combination with pembrolizumab, an anti-PD-1 antibody. Early data suggest that SNX281 can enhance anti-tumor immunity, potentially overcoming resistance to checkpoint inhibitors in some cancers. SNX281’s mechanism involves activating STING, which leads to the production of type I interferons and pro-inflammatory cytokines, ultimately stimulating the infiltration of immune cells into the tumor microenvironment. This process could help convert ‘cold’ tumors into ‘hot’ tumors, making them more responsive to immunotherapy (41).

#### 3.2.2 HG-381

HG-381 is a non-CDN STING agonist developed by HitGen, currently in Phase I clinical trials (ClinicalTrials.gov-NCT04998422). This small molecule is being tested for its ability to treat advanced solid tumors. While specific clinical data is still emerging, HG-381 has shown potential in preclinical models to induce a strong immune response by activating STING, leading to the production of type I interferons and other cytokines that can suppress tumor growth. The ongoing clinical trials aim to determine the safety, optimal dosing, and preliminary efficacy of HG-381 in cancer patients (42).

#### 3.2.3 GSK3745417

GSK3745417 is a non-CDN STING agonist developed by GlaxoSmithKline, currently in Phase I clinical trials (ClinicalTrials.gov-NCT03843359). This agonist is being evaluated for its efficacy in treating advanced solid tumors. GSK3745417 works by directly binding to and activating the STING receptor, leading to the production of interferons and other immune-modulatory cytokines. The ongoing trials are focused on assessing the safety, tolerability, and early signs of efficacy, both as a monotherapy and potentially in combination with other immunotherapies. Early data suggest that GSK3745417 has a manageable safety profile, with potential for inducing anti-tumor immune responses (43).

#### 3.2.4 E-7766

E-7766 is a non-CDN STING agonist developed by Eisai, currently in Phase I clinical trials (ClinicalTrials.gov-NCT04144140). This drug is being tested in patients with advanced solid tumors and lymphomas. E-7766 is designed to activate the STING pathway, thereby stimulating the immune system to attack cancer cells. Early clinical studies are assessing the safety, tolerability, and preliminary efficacy of E-7766. The initial data indicate that E-7766 can induce immune activation with a tolerable safety profile, although further studies are needed to determine its therapeutic potential in combination with other cancer treatments (44, 45).

### 3.3 CDN-infused exosomes

CDN-loaded exosome STING agonists represent an innovative approach to cancer immunotherapy, combining the potency of cyclic dinucleotide (CDN) STING agonists with the targeted delivery capabilities of exosomes. Exosomes are small, naturally occurring vesicles that can be engineered to carry therapeutic molecules, such as CDNs, directly to specific cells, including immune cells within the tumor microenvironment. This method enhances the effectiveness of STING activation while potentially reducing systemic toxicity.

exoSTING is a prime example of a CDN-loaded exosome STING agonist, developed by Codiak BioSciences. This therapy is designed to enhance the delivery and activation of the STING pathway within tumors, leveraging the natural properties of exosomes to improve the targeting and uptake of CDNs by immune cells. exoSTING is currently being evaluated in Phase I/II clinical trials for advanced solid tumors (ClinicalTrials.gov-NCT04592484). The drug is administered intratumorally, with the aim of directly stimulating the STING pathway within the tumor microenvironment. The exosome-based delivery system allows for a localized and potent activation of STING, leading to robust immune responses characterized by increased infiltration of T cells and other immune effectors into the tumor. The ongoing clinical trials are focused on further characterizing the safety and efficacy of exoSTING, as well as exploring its potential in combination with other immunotherapies, such as checkpoint inhibitors. The preliminary results are promising, suggesting that exoSTING could become a valuable addition to the arsenal of STING-based cancer therapies, particularly for tumors that are resistant to conventional treatments. In addition, the ability of exoSTING to focus its activity within the tumor microenvironment while minimizing systemic exposure may reduce the risk of side effects, making it an attractive option for enhancing the therapeutic index of STING agonists (46).

### 3.4 Engineered bacteria vectors

Engineered bacteria vectors represent a novel and innovative approach to cancer immunotherapy, where genetically modified bacteria are used to deliver therapeutic agents directly to the tumor microenvironment. These bacteria are designed to produce and release STING agonists within the tumor, thereby activating the STING pathway in situ. This method leverages the natural ability of bacteria to colonize tumors, providing a targeted and sustained activation of the immune system against cancer cells.

SYNB1891 is a leading example of an engineered bacteria vector designed to activate the STING pathway. Developed by Synlogic, SYNB1891 is a live, engineered strain of the probiotic bacterium of Escherichia coli Nissle 1917, which has been modified to produce cyclic di-GMP, a potent STING agonist. This STING agonist is released directly within the tumor microenvironment, where it can trigger an immune response by activating the STING pathway in local immune cells.

SYNB1891 is currently in Phase I clinical trials for patients with advanced solid tumors (ClinicalTrials.gov-NCT04167137). The drug is administered intratumorally, allowing the bacteria to colonize the tumor and produce the STING agonist directly where it is needed. The Phase I trials are primarily focused on evaluating the safety and tolerability of SYNB1891, as well as its ability to induce an immune response. The intratumoral administration of SYNB1891 has led to localized immune activation, characterized by increased production of type I interferons and other pro-inflammatory cytokines within the tumor. These immune responses are associated with enhanced infiltration of T cells into the tumor, suggesting that SYNB1891 may help convert “cold” tumors, which are typically resistant to immunotherapy, into “hot” tumors that are more responsive to immune-based treatments. Furthermore, the use of engineered bacteria allows for sustained release of STING agonists within the tumor microenvironment, offering prolonged immune activation without the need for repeated systemic dosing (47).

In addition to its use as a monotherapy, SYNB1891 is also being evaluated in combination with immune checkpoint inhibitors, such as atezolizumab (anti-PD-L1). The rationale for this combination is that the STING-mediated immune activation induced by SYNB1891 could enhance the effectiveness of checkpoint inhibitors, particularly in tumors that have previously shown resistance to such therapies. The ongoing clinical trials aim to further characterize the immune responses induced by SYNB1891 and to assess its potential as a component of combination therapies for cancer. The preliminary results are promising, indicating that engineered bacteria vectors like SYNB1891 could offer a new and effective approach to cancer immunotherapy (48).

### 3.5 Hybrid structures of small molecules-nucleic acids

Small molecule–nucleic acid hybrids represent an emerging class of STING agonists designed to combine the stability and pharmacokinetic properties of small molecules with the specificity and functionality of nucleic acids. These hybrids aim to enhance the activation of the STING pathway while improving drug delivery and minimizing off-target effects. This innovative approach seeks to harness the advantages of both molecular types to create more effective and targeted cancer therapies.

SB 11285 is a leading example of a small molecule–nucleic acid hybrid STING agonist, developed by Spring Bank Pharmaceuticals. SB 11285 is designed to activate the STING pathway more effectively than traditional small molecules by leveraging its hybrid structure, which combines a potent small molecule with a nucleic acid component. By combining the stability of a small molecule with the specificity of a nucleic acid, SB 11285 aims to overcome the limitations of conventional STING agonists, ensuring better pharmacokinetic properties and more targeted immune activation. SB 11285 is currently in Phase I/II clinical trials for patients with solid tumors and hematologic malignancies (ClinicalTrials.gov-NCT04096638). The ongoing trials are also exploring SB 11285 in combination with immune checkpoint inhibitors, such as atezolizumab (anti-PD-L1). The rationale behind this combination is that SB 11285’s activation of the STING pathway could enhance the effectiveness of checkpoint inhibitors by increasing the immune system’s ability to recognize and attack tumor cells. Early results suggest that the combination therapy may improve outcomes in patients with tumors that are otherwise resistant to checkpoint inhibitors alone. The trials aim to further assess the efficacy of SB 11285, particularly its ability to induce durable responses and improve survival outcomes in cancer patients. As a small molecule–nucleic acid hybrid, SB 11285 represents a promising new approach in the development of STING-based therapies, potentially offering enhanced efficacy and safety over traditional STING agonists (49, 50).

### 3.6 Undisclosed type

The “Undisclosed Type” category includes STING agonists whose precise molecular mechanisms or structures have not been fully disclosed to the public. These agonists are often in early stages of development, with companies keeping details confidential for strategic reasons. Despite the lack of detailed structural information, these agents are advancing through clinical trials and show potential in activating the STING pathway for cancer immunotherapy.

#### 3.6.1 MK-2118

MK-2118 is a STING agonist developed by Merck, currently in Phase I clinical trials (ClinicalTrials.gov-NCT03249792). While the exact molecular structure of MK-2118 has not been publicly revealed, it is known that the drug is being tested in patients with solid tumors, particularly in combination with pembrolizumab, an anti-PD-1 antibody. Early clinical data suggest that MK-2118 has a favorable safety profile and can enhance the anti-tumor effects of pembrolizumab. MK-2118 is administered intratumorally, ensuring that the STING agonist is delivered directly to the tumor microenvironment, which helps maximize immune activation locally while reducing potential systemic side effects. Furthermore, its ability to activate the STING pathway may help improve the immune system’s recognition of tumors that are resistant to conventional immunotherapies. The combination therapy aims to stimulate a stronger immune response against tumors that are resistant to checkpoint inhibitors alone (51).

#### 3.6.2 XMT-2056

XMT-2056 is another STING agonist with an undisclosed mechanism, developed by Mersana Therapeutics. XMT-2056 is currently in Phase I clinical trials, focusing on patients with advanced solid tumors (ClinicalTrials.gov-NCT05514717). This drug is administered intravenously and is designed to target tumors with a high degree of precision. Although detailed clinical data are still pending, XMT-2056 is being investigated both as a monotherapy and in combination with other cancer treatments, including immune checkpoint inhibitors. The goal of these studies is to assess the drug’s safety, tolerability, and preliminary efficacy in inducing an anti-tumor immune response.

The secrecy surrounding these agents adds an element of intrigue, as the exact mechanisms by which they activate the STING pathway remain speculative. However, the ongoing clinical trials are crucial in determining their potential efficacy and safety profiles. The early-stage results for both MK-2118 and XMT-2056 are promising, suggesting that these undisclosed-type STING agonists could become significant players in the field of cancer immunotherapy (52, 53).

While the STING agonists discussed in this review encompass a diverse range of structures—spanning CDN analogs, small molecule agonists, nucleic acid hybrids, and engineered bacterial vectors—these compounds share several key functional characteristics. Most notably, they all target the STING pathway by engaging the STING protein to initiate immune signaling. CDN analogs, such as MK-1454 and exoSTING, closely mimic natural cyclic dinucleotides, binding to the same pocket on the STING protein that recognizes endogenous STING activators. Non-CDN agonists, including SNX281 and SB 11285, achieve similar activation through alternative molecular scaffolds, ensuring they can engage STING without mimicking the natural ligands. Despite these structural differences, the shared objective of these agonists is to stimulate type I interferon production and activate pro-inflammatory cytokines, leading to enhanced immune infiltration into the tumor microenvironment. By modulating the immune response in this way, STING agonists can convert ‘cold’ tumors into ‘hot’ tumors, making them more susceptible to immunotherapies.

To comprehensively showcase the current research progress of STING agonists in cancer immunotherapy, we have summarized the STING agonists at various stages of clinical development (**Table 1**). These agonists encompass a variety of chemical types, including cyclic dinucleotide (CDN) analogs, non-CDN chemotypes, CDN-loaded exosomes, and engineered bacterial vectors. Detailed information on each agonist's developing company, clinical trial phase, administration methods, and whether they are used in combination with other immunotherapies is provided. Through this table, readers can gain a clear understanding of the current research hotspots and clinical advancements of STING agonists.

**Table 1:** Summary of STING agonists currently in clinical trials, including their chemical structures, target phases, indications, combination therapies, and clinical outcomes.

Drug Name	Chemical Structure	Target Phase	Indication	Combination Therapies	Clinical Outcome
MK-1454	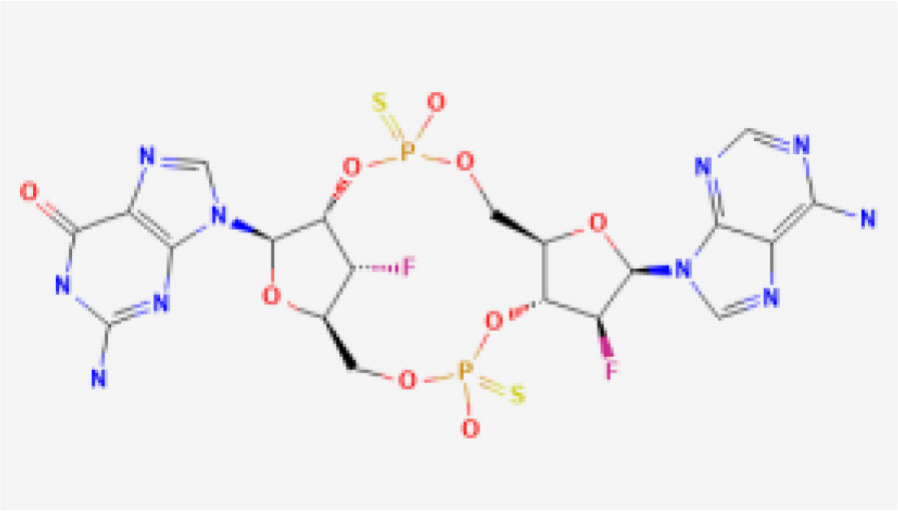	Phase I/II	Solid Tumors, Lymphomas	Pembrolizumab (anti-PD-1)	Early results show safety and potential efficacy
ADU-S100 (MIW815)	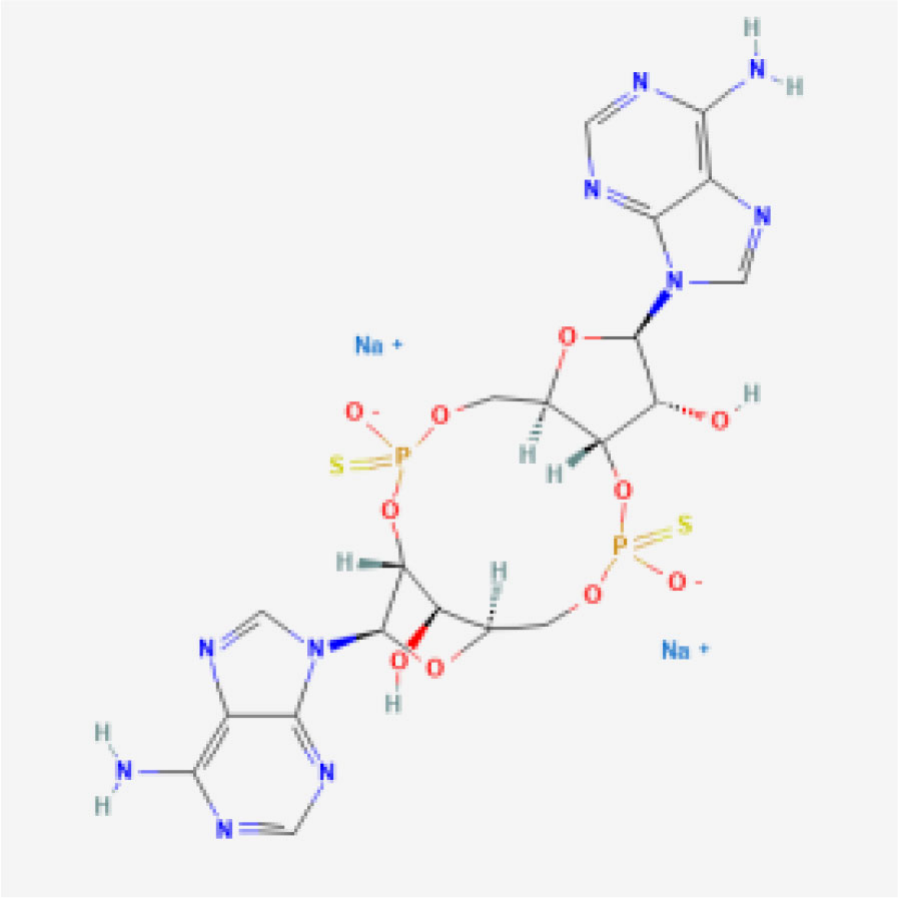	Phase I/II	Solid Tumors, Lymphomas	Spartalizumab (anti-PD-1)	Encouraging immune response in combination with PD-1 blockade
TAK-676	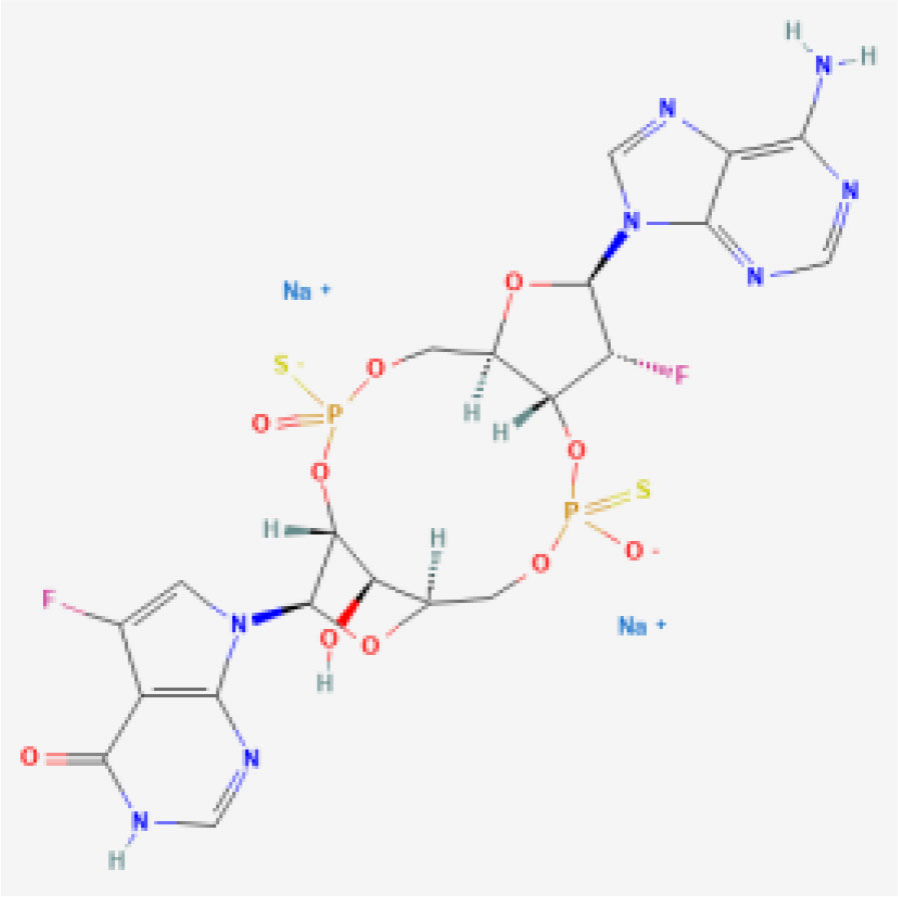	Phase I/II	Advanced Solid Tumors, Lymphomas	± Pembrolizumab (anti-PD-1)	Ongoing, early data pending
BI-1387446	The structure has not been made public, it belongs to the CDN small molecule agonist class	Phase I	Advanced Cancers	± Ezabenlimab (anti-PD-L1)	Ongoing, early data pending
BMS-986301	The structure has not been made public, it belongs to the CDN small molecule agonist class	Phase I	Advanced Cancers	None currently reported	Ongoing, early data pending
E7766	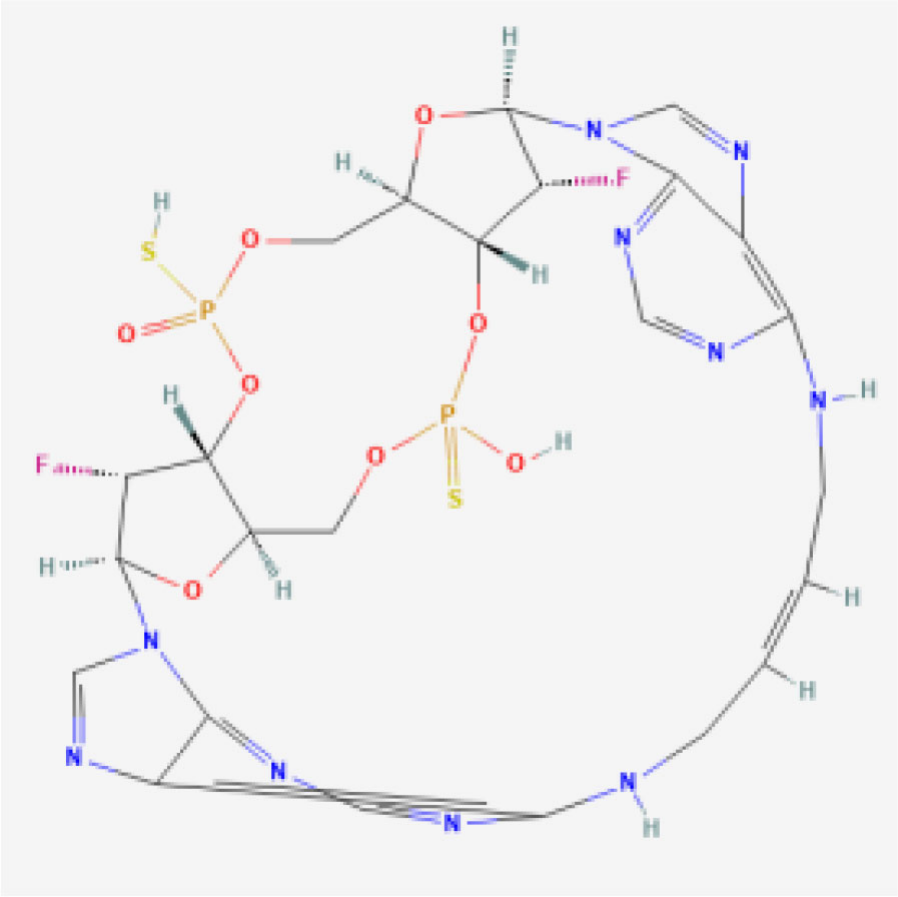	Phase I	Advanced Solid Tumors, Lymphomas	None currently reported	Ongoing, early data pending
SNX281	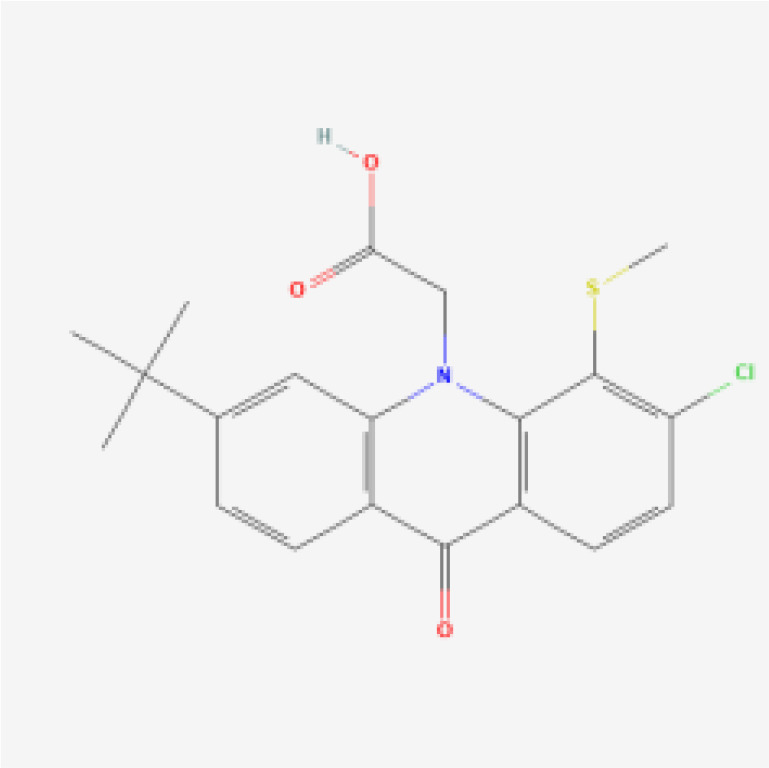	Phase I	Advanced Solid Tumors	None currently reported	Ongoing, early data pending
HG-381	The structure has not been made public, it belongs to the non-CDN small molecule agonist class	Phase I	Advanced Solid Tumors	Monotherapy	Early-stage, shows potential
GSK3745417	The structure has not been made public, it belongs to the non-CDN small molecule agonist class	Phase I	Advanced Solid Tumors	None currently reported	Ongoing, early data pending
exoSTING	The structure has not been made public, it belongs to the CDN-loaded exosome class	Phase I/II	Advanced Solid Tumors	Monotherapy	Completed, shows promising results
SYNB1891	The structure has not been made public, it belongs to the engineered bacteria vector class	Phase I	Advanced Solid Tumors	± Atezolizumab	Completed, data pending
SB 11285	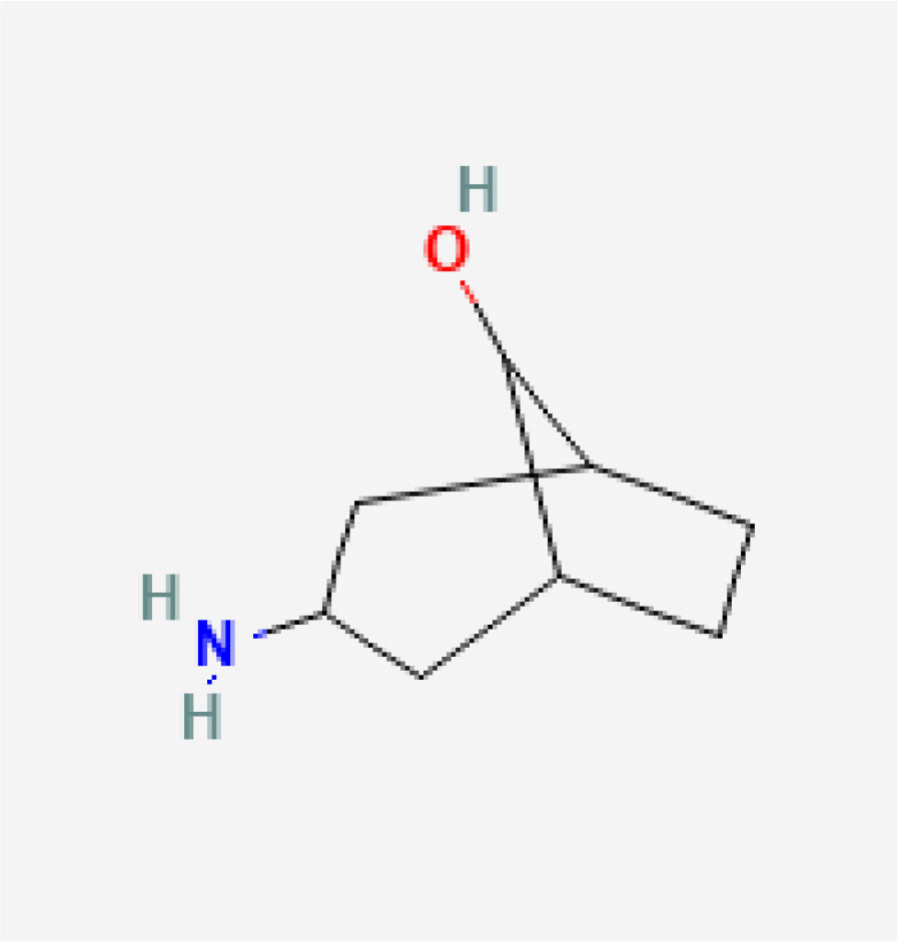	Phase I/II	Solid Tumors, Hematologic Malignancies	Potential future combination with checkpoint inhibitors	Early-stage results showing promise in safety and immunogenicity
MK-2118	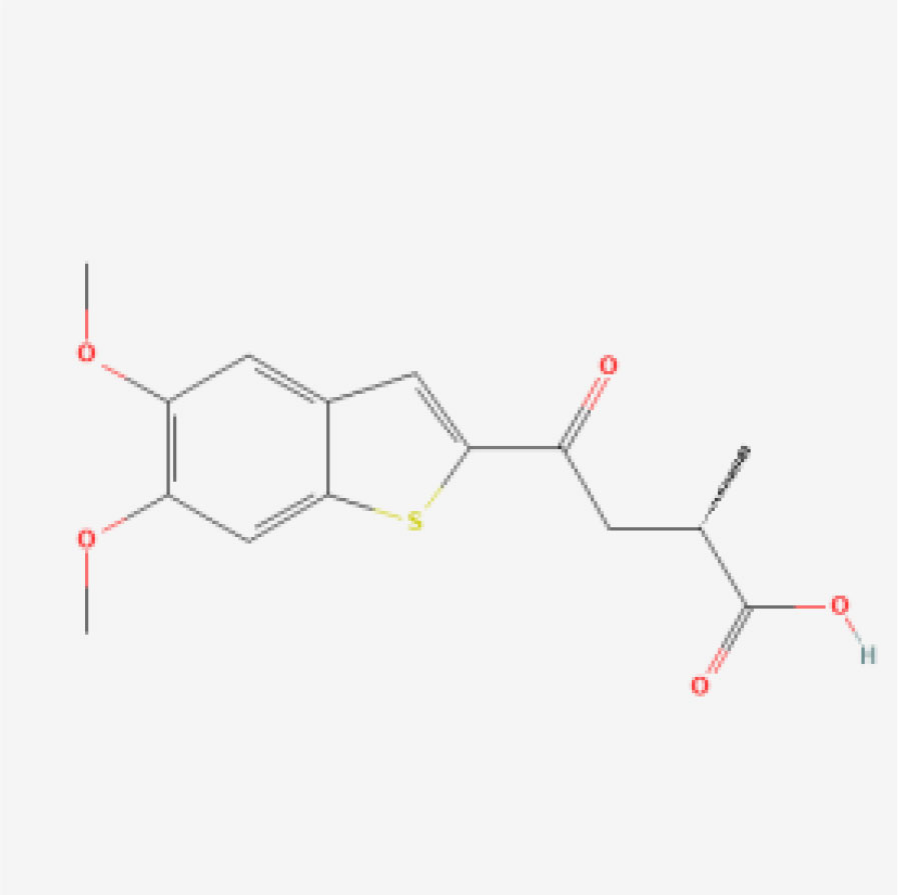	Phase I	Solid Tumors	± Pembrolizumab	Completed, shows potential
XMT-2056	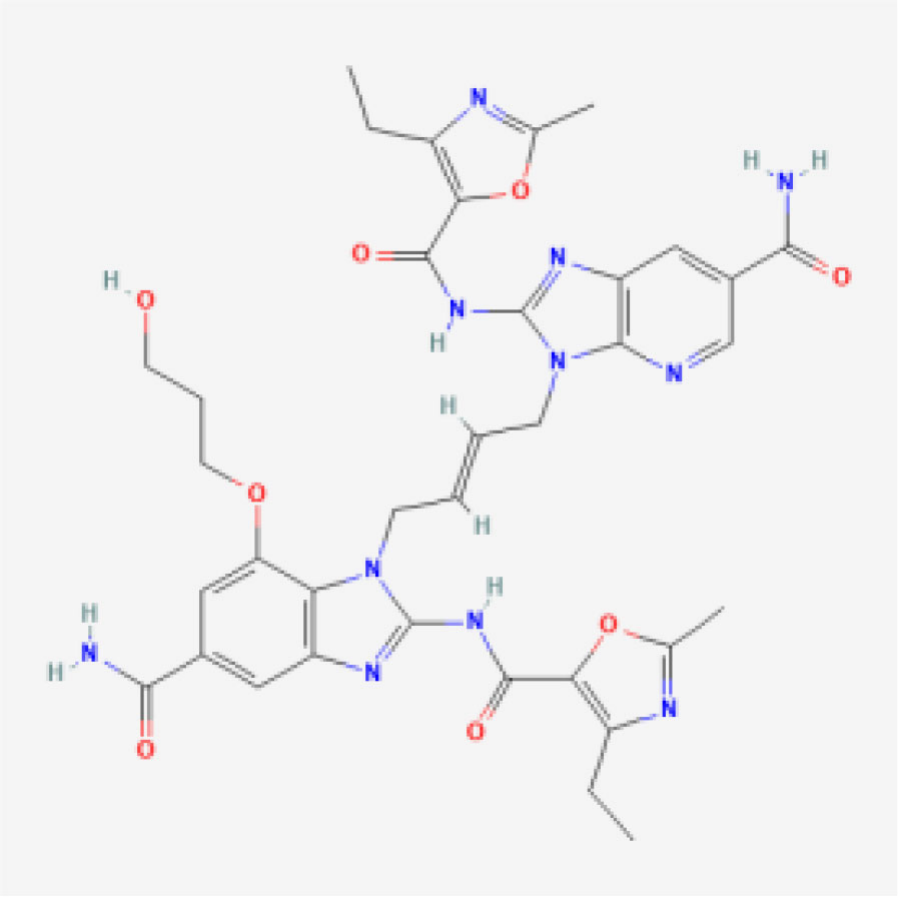	Phase I	Advanced Solid Tumors	Monotherapy	Ongoing, early data pending

## 4 Challenges and limitations

While STING agonists represent a promising avenue for cancer immunotherapy, several challenges and limitations must be addressed to fully realize their potential. These challenges span from biological complexities to clinical application hurdles, each requiring careful consideration in the development and deployment of STING-based therapies.

Managing the toxicity of STING agonists is a significant challenge. Activation of the STING pathway can lead to the excessive production of pro-inflammatory cytokines, including type I interferons (e.g., IFN-α, IFN-β) and non-type I interferons such as IFN-γ, causing severe side effects like fever, chills, and cytokine release syndrome (CRS). The risk is higher with systemic administration, making it crucial to optimize dosing strategies and develop more selective STING activators to minimize off-target effects (54). Tumor heterogeneity and response variability present major challenges for STING agonists. Tumors differ in genetic makeup and immune environment, leading to inconsistent responses. “Cold” tumors with low immune cell infiltration may not respond well due to insufficient STING ligands or necessary immune cells. Identifying predictive biomarkers is essential to select patients who will benefit most from STING-based therapies (55). Effective delivery and targeting of STING agonists remain challenging. Intratumoral injections, while effective, are not feasible for all tumors, and systemic delivery risks widespread immune activation and toxicity (56, 57). Experimental methods like nanoparticles, exosomes, and engineered bacteria are being explored to improve targeting and minimize systemic exposure, but further research is needed to confirm their efficacy and safety (58, 59).

Resistance to STING agonists is a significant concern in cancer therapy. Tumors may downregulate STING expression, mutate pathway components, or alter the microenvironment to evade immune responses. Understanding these mechanisms is crucial for developing combination therapies, such as pairing STING agonists with checkpoint inhibitors or chemotherapies, to enhance efficacy and prevent resistance (60). Finally, Regulatory and manufacturing challenges are significant for STING agonists, especially those with novel mechanisms or delivery systems. Rigorous testing is needed to meet regulatory standards, and the complexity of therapies involving engineered bacteria or exosomes complicates manufacturing, scalability, and quality control. Ensuring consistent production and stability is crucial for successful clinical translation (3, 61, 62).

In some types of cancers, the STING pathway may be deficient due to genetic mutations, epigenetic silencing, or functional suppression within the tumor microenvironment (63). Repairing the STING pathway in these cases is a significant challenge, but several strategies are under investigation. Gene therapy approaches, such as using CRISPR or viral vectors, could be employed to repair mutations in the STING gene or other components of the pathway, restoring STING functionality. Alternatively, combination therapies that pair STING agonists with immune checkpoint inhibitors or DNA damage response inhibitors may enhance immune activation even in tumors with partial STING deficiency. Epigenetic therapies, which reverse silencing of STING-related genes, are also being explored to restore STING pathway signaling. While these approaches hold promise, further clinical studies are needed to determine their viability and effectiveness in repairing STING pathway deficiencies (64).

## 5 Conclusion

STING agonists have emerged as a promising class of agents in cancer immunotherapy, capable of initiating a robust immune response through the activation of the STING pathway. These agents have demonstrated potential in early-phase clinical trials, particularly when combined with immune checkpoint inhibitors, offering hope for treating tumors that are resistant to conventional therapies. The ability of STING agonists have the potential to convert ‘cold’ tumors, which lack immune cell infiltration, into ‘hot’ tumors that are more responsive to immunotherapy by promoting the production of chemokines such as CCL5, CXCL9, and CXCL10, which recruit immune cells like T cells and NK cells to the tumor microenvironment, highlights their transformative potential in cancer treatment.

However, several challenges must be addressed to fully realize the clinical potential of STING agonists. Managing the toxicity associated with systemic immune activation, ensuring effective delivery to tumor sites, and overcoming tumor heterogeneity and resistance mechanisms are critical hurdles. Additionally, the development and manufacturing of STING agonists, especially those involving novel delivery systems like nanoparticles and engineered bacteria, pose significant regulatory and quality control challenges.

Despite these obstacles, the future of STING agonists in cancer therapy remains bright. Continued research into optimizing delivery methods, identifying predictive biomarkers, and developing combination therapies will be key to overcoming current limitations. As our understanding of the STING pathway deepens, these agents could become integral components of cancer treatment, offering new hope to patients with difficult-to-treat malignancies. The next few years will be crucial in determining whether STING agonists can transition from experimental therapies to widely accepted clinical options, potentially revolutionizing the landscape of cancer immunotherapy.
